# Perceived Consequences of Post-COVID-19 and Factors Associated with Low Life Satisfaction

**DOI:** 10.3390/ijerph192215309

**Published:** 2022-11-19

**Authors:** Elisabeth Ekstrand, Christina Brogårdh, Iben Axen, Agneta Malmgren Fänge, Kjerstin Stigmar, Eva Ekvall Hansson

**Affiliations:** 1 Department of Health Sciences, Lund University, 221 85 Lund, Sweden; 2 Department of Hand Surgery, Skåne University Hospital, 205 02 Malmö, Sweden; 3 Department of Neurology, Rehabilitation Medicine, Memory Disorders and Geriatrics, Skåne University Hospital, 221 40 Lund, Sweden; 4 Intervention and Implementation Research for Worker Health, Institute of Environmental Medicine, Karolinska Institutet, Nobels v. 13, 171 77 Stockholm, Sweden; 5 Ear-Nose- and Throat Department, Skåne University Hospital, 221 40 Lund, Sweden

**Keywords:** activities of daily living, COVID-19, health, life satisfaction, physical activity, post-COVID-19 condition, work

## Abstract

A significant number of individuals experience post-COVID-19 symptoms, but knowledge of perceived consequences and life satisfaction is lacking. Here, we investigate perceived consequences regarding everyday life, health, physical activity and work post-COVID-19 and factors associated with low life satisfaction. A total of 766 people (mean age 48; 672 women) experiencing post-COVID-19 symptoms at least two months after infection (mean 13 months) responded to an online survey. A majority (≥77%) perceived physical fatigue, mental fatigue, dizziness, reduced work ability, low life satisfaction and a reduced level of aerobic capacity. In the final logistic regression model (Nagelkerke R Square 0.296, *p* < 0.001), poor work ability was the most important factor for perceiving low satisfaction with life (Odds ratio 3.369, 95% CI 2.040–5.565, *p* < 0.001, Nagelkerke R Square 0.177). Reduced aerobic capacity, fatigue and living in a city also increased the odds of low life satisfaction. As people with post-COVID-19 report several long-term consequences, this suggests that there is a need for targeted care for this group. The results of this study can serve as guidance for healthcare authorities regarding important long-term consequences that should be considered in rehabilitation programs directed toward post-COVID-19.

## 1. Introduction

According to the World Health Organization (WHO) there have now been about 600 million confirmed cases of COVID-19 worldwide [1], and the virus still continues to infect people. A feature of COVID-19 that differs from other respiratory infections is the multi-system symptomatology and long-term sequelae [2]. Most persons who develop COVID-19 fully recover, but current research suggests that approximately 10 to 20% experience a variety of mid- and long-term symptoms after their initial illness, known as post-COVID-19 condition [1].

Post-COVID-19 is defined as a condition that occurs in individuals with a history of probable or confirmed SARS-CoV-2 infection, with symptoms that last for at least two months and cannot be explained by an alternative diagnosis [3]. The mechanisms behind post-COVID-19 are not fully understood, but it has been suggested that they are associated with dysregulation of the immune and autonomic nervous systems due to viral injury, oxidative stress, immunologic abnormalities and inflammatory damage [4]. Post-COVID-19 can involve a range of symptoms, such as fatigue and muscle weakness, chest and muscle pain, shortness of breath, anosmia/ageusia, fever, cognitive dysfunction/brain fog, headache, tachycardia and intestinal disorders [5,6,7]. The symptoms can persist from the acute COVID-19 infection, but new symptoms may also occur after initial recovery and can fluctuate or relapse over time [3].

The severity of the acute COVID-19 infection, comorbidities and advanced age have been identified as risk factors for post-COVID-19 [5,8,9]. However, it has been shown that it also affects younger persons with a mild acute illness that did not need hospital or intensive care [8,10,11,12]. Studies, including mostly younger (<60 years) and non-hospitalized persons, have reported fatigue as the most common post-COVID-19 symptom [13,14,15,16]. A longitudinal online survey investigating post-COVID-19 in mostly non-hospitalized persons from 56 countries showed that over 90% experienced symptoms seven months after the acute infection, and about 70% of unrecovered persons had not returned to previous levels of work due to their illness [13]. Cross-sectional studies have reported reduced physical activity levels, cognitive impairments and limitations in daily activities after milder COVID-19 infections [14,15,16]. Thus, COVID-19 can lead to long-term disability, which may have a significant impact on physical and mental health and on the ability to manage everyday activities and work.

As long-term disability can impact life satisfaction negatively [17], it is important to study how persons with post-COVID-19 perceive the consequences of the disease and how it affects their life satisfaction. Perceived life satisfaction is related to concepts such as well-being, contentment and happiness and is also affected by expectations and aspirations as well as the subjective appraisal of the extent to which these are being met [18,19]. The level of life satisfaction can be assessed as the perceived overall life satisfaction and also in relation to different aspects of life such as provision, leisure, close relations and health [20,21]. Previous studies have shown that various factors such as age, family situation, educational level, employment situation, residential area and comorbidities can affect the level of perceived life satisfaction [21,22,23,24,25,26].

Currently, there is a lack of knowledge of perceived consequences and life satisfaction with post-COVID-19. Increased knowledge can improve the ability of welfare authorities and the healthcare system to support these people. The aim of the study was, therefore, to investigate perceived consequences regarding everyday life, health, physical activity and work post-COVID-19 and the factors associated with low life satisfaction.

## 2. Materials and Methods

### 2.1. Study Design

This study had a cross-sectional design and was part of a larger project on life after COVID-19 (The LAC project) investigating different aspects of the long-term consequences of COVID-19 and their impact on life.

### 2.2. Recruitment and Participants

Recruitment of participants was conducted by means of an announcement on social media, posted between the 21 October and the 13 November 2021. Persons 18 years or older, able to read and understand Swedish and having had a COVID-19 infection with remaining symptoms, were invited to participate. The current study included people with remaining symptoms for at least 2 months after the acute infection.

A Facebook page with information about the project was targeted to persons in the three most populated regions of Sweden (Stockholm, Gothenburg and Skåne) but could also be shared with users outside these areas. The link to the invitation was also posted on Instagram and Twitter. The project webpage was hosted at Lund University and included general project information, a participant information sheet, and a link to the survey. The survey was open until 12 February 2022, which resulted in a total of 867 persons participating in the survey. Of those, 52 persons did not meet the inclusion criteria on remaining post-COVID-19 symptoms for at least 2 months, and 49 persons were excluded due to not completing the mandatory background questions, giving a total of 766 participants who were included in the study.

### 2.3. Data Collection

Data collection was completed using REDCap (Research Electronic Data Capture), a secure, web-based application designed to support data capture for research studies [27,28]. Outcome measures were chosen based on recent descriptions of symptoms and potential consequences of post-COVID-19 [29].

The online survey included sociodemographic questions regarding age, sex (man or woman), family situation (single, married/cohabiting or partner but not cohabiting), residential community characteristics (city, town or village) and level of education (primary school, secondary school or higher education), provision (work, student grants, benefits due to sickness, unemployment or social security issues or other sources of income) and comorbidities (yes or no). The participants answered questions on the acute COVID-19 infection (onset, symptoms and need for hospital care) and post-COVID-19 condition (duration and symptoms). They also responded to questionnaires on the perceived consequences of COVID-19.

### 2.4. Questionnaires on Perceived Consequences of COVID-19

Physical fatigue was assessed with the Fatigue Severity Scale (FSS) [30], which has demonstrated adequate validity and reliability (Cronbach’s alpha 0.93–0.96; intraclass correlation coefficient (ICC) 0.84; Kappa coefficient 0.75) in various diagnoses [31,32,33]. The FSS consists of 9 statements concerning the impact of fatigue on daily life that are scored from 1 (strongly disagree) to 7 (strongly agree). The total score of the FSS ranges from 1 to 7 (mean of the 9 statements), where a greater score indicates more fatigue and a cut-off score of ≥4 signifies physical fatigue [34].

Mental fatigue was assessed with the Mental Fatigue Scale (MFS) [35]. The MFS was developed to capture mental fatigue regardless of illness and has demonstrated high internal consistency (Cronbach’s alpha 0.94) [35]. It includes 15 items scored from 0 (normal function) to 3 (maximal symptoms). The total score is calculated as the sum of items 1–14, and item 15 provides additional information on daytime variation of symptoms. A sum score ≥10.5 indicates mental fatigue [36].

Perceived dizziness and balance impairment related to COVID-19 were assessed by a single question (yes or no) that has been used in previous studies of dizziness [37].

Level of dependence on another person in daily activities (ADL) was assessed by the ADL Staircase [38] that has shown acceptable construct validity and internal consistency (Cronbach’s alpha 0.88) in various age groups [39]. The ADL Staircase comprises 5 personal (P-ADL) and 5 instrumental daily activities (I-ADL) that are rated on a 4-graded scale as independent without difficulties (0), independent with difficulties (1), partly dependent (2) or dependent (3). The subscores of P-ADL and I-ADL range from 0 to 15, and the total score ranges from 0 to 30.

Current perceived aerobic capacity was assessed by the Rating of Perceived Capacity scale (RPC) [40]. RPC is valid and considered a valuable tool for the estimation of aerobic capacity in research studies [40,41]. The RPC is based on metabolic equivalents (METs) that are linked to physical activities on a progressive scale. The most strenuous activity that can be sustained for at least 30 min is rated from 1 (sit) to 20 (elite aerobic training). The maximal value (elite aerobic training) is different for the two genders, 18 for women and 20 for men.

Work ability was measured with the Work Ability Score (WAS) [42]. The WAS has been proven valid and reliable (ICC 0.89) for assessing work ability in research [42,43]. The WAS is based on the perceived current work ability in relation to lifetime best, ranges from 0 to 10 and can be categorized as poor (0–5 points), moderate (6–7 points), good (8–9 points) or excellent (10 points) [44].

Life satisfaction was rated using the Life Satisfaction Questionnaire (LiSat-11) [21,22]. The LiSat-11 is valid and reliable (Kappa coefficient 0.59–0.97), and reference values are available based on ratings of 2533 Swedish individuals aged 18 to 65 years [22,45,46]. The questionnaire includes 11 items and assesses how satisfied an individual is with overall life satisfaction, Life as a whole (item 1) and with 10 domain-specific items regarding vocation, economy, leisure, contacts with friends and acquaintances, intimacy, activities of daily living (ADL), family life, partnership/relationship, physical health and psychological health. The items are rated as very dissatisfying (score 1), dissatisfying (score 2), rather dissatisfying (score 3), rather satisfying (score 4), satisfying (score 5) and very satisfying (score 6). The score can be dichotomized into low life satisfaction (score 1–4) and high life satisfaction (score 5–6) [22]. In the current study, the participants also reported if they experienced Life as a whole as deteriorated, unchanged or improved compared to before the COVID-19 infection and if they felt that the change was due to COVID-19.

### 2.5. Statistical Analyses

Statistical analyses were performed with SPSS version 28.0 (IBM Corporation, Armonk, New York, NY, USA). Probability values less than 0.05 were considered statistically significant. For descriptive data, means (standard deviations, SD), frequencies and medians (interquartile ranges, IQR and maximum and minimum values) were calculated.

The proportion of participants with low life satisfaction (LiSat-11 score 1–4) was presented for each item of LiSat-11 and compared to the proportion of satisfied persons in the Swedish reference sample [22] by means of the One Sample Proportion Test.

The association of potential explanatory factors with life satisfaction was investigated with logistic regression analyses. Life as a whole (item 1 in LiSat-11) was used as an overall measure of perceived life satisfaction (dependent variable) and dichotomized into low and high life satisfaction. Potential explanatory independent variables added in the regression building were sociodemographic factors that, in previous studies, have been shown to impact life satisfaction [21,22,23,24,25,26] and potential explanatory factors of consequences related to post-COVID-19. The sociodemographic factors included in the model building were: age, sex (man vs. woman), family situation (single vs. married/partner), educational level (lower vs. higher education), provision (not working vs. working), residential community (city vs. town/village) and comorbidities (no vs. yes). Consequences related to post-COVID-19 were: physical fatigue (no vs. yes), mental fatigue (no vs. yes), dizziness (no vs. yes), balance impairment (no vs. yes), ADL (ADL staircase score), aerobic capacity (RPC score) and work ability (moderate–excellent ability vs. poor ability).

The associations with overall life satisfaction (i.e., Life as a whole) were evaluated for each explanatory factor separately using univariate logistic regression analyses. The odds ratio, 95% confidence interval (CI), explanatory value (Nagelkerke R Square) and *p*-value were calculated. A generous inclusion criterion (*p* ≤ 0.20) was used to ensure that no potential variable was omitted in the following multivariate regression analysis. The variable with the lowest p-value (if ≤ 0.20) from the univariate analysis was included in the model. Thereafter, the other factors were tentatively added, one at a time. The model with the highest explanatory value and the two independent variables with the lowest *p*-values (if both *p* ≤ 0.20) were kept. Thereafter, the remaining factors were again added, one at a time, and the model with the highest explanatory value and variables with the lowest *p*-values (if *p* ≤ 0.20) were retained. Thus, in each step, one variable was added to the model. This procedure was continued as long as the *p*-value of all the included variables in the model was *p* ≤ 0.20 and the explanatory value increased.

### 2.6. Ethics

All participants gave their consent to participate in the study by clicking on a link that directed them to the online survey. The study was approved by the Swedish Ethical Review Authority (Dnr 2020-02776), and the principles of the Declaration of Helsinki were followed.

## 3. Results

There were 766 persons who completed the survey, but as the participants could choose not to answer a question/questionnaire, the number of answers varies (see detailed information for each variable in the tables).

### 3.1. Characteristics of the Participants

Most participants were middle-aged (mean 48 years, SD 10), women (89%), highly educated (72%), working (69%) and approximately equally distributed in terms of their residential community (living in a city, town or village), see Table 1.

Thirty-nine percent reported comorbidities such as asthma (27%), thyroid dysfunction (19%), allergies (12%) and hypertension (11%). A majority developed their acute COVID-19 infection during the second wave (autumn and winter of 2020–2021), and most persons were not in need of hospital care (89%). The most commonly reported acute COVID-19 symptoms were fatigue (88%), fever (74%), headache (73%) and anosmia/ageusia (67%). The remaining COVID-19 symptoms (for at least two months) were fatigue (79%), joint and muscle pain (45%), anosmia/ageusia (42%), dyspnea (39%), chest pain (35%) and cough (18%). The mean duration of post-COVID-19 was 13 months (SD 5), Table 1.

### 3.2. Perceived Consequences of COVID-19

According to the questionnaires on perceived consequences of COVID-19, a majority of the participants experienced physical fatigue (85%), mental fatigue (84%), dizziness (84%) and balance impairments (56%); see Table 2. The median perceived aerobic capacity measured by the RPC was 5 (IQR 3–7), i.e., walking or cycling slowly was the most strenuous activity that could be sustained for at least 30 min. Most persons perceived no difficulties in ADL, but 78% perceived reduced work ability (WAS) (poor or moderate) compared to their lifetime best.

Overall life satisfaction (i.e., Life as a whole) showed a median of 4 (IQR 3–4), whereof 77% of the participants perceived low satisfaction with Life as a whole (Table 3). For 87%, Life as a whole was experienced as deteriorated compared to before COVID-19, and almost all persons (98%) answered that the deterioration was due to or partly due to COVID-19.

For the domain-specific items (2–11), a large proportion of the participants perceived low satisfaction with Physical health (91%) and Leisure (89%), and a majority experienced low satisfaction with the other items (51–79%) except for ADL (33%) and Partner relationship (48%). Compared to the Swedish reference values [22], a significantly higher proportion of persons in our sample perceived low satisfaction with Life as a whole and all domain-specific items (*p* < 0.001) except for Economy (*p* = 0.096); see Table 3.

### 3.3. Factors Associated with Life as a Whole

Work ability had the strongest univariate association with low satisfaction of Life as a whole (Odds ratio 6.255, 95% CI 3.978–9.837, *p* < 0.001) (see Table 4), and the factors of aerobic capacity, physical fatigue, mental fatigue, balance impairment, dizziness, ADL, provision, residential community and family situation also fulfilled the criteria (*p* ≤ 0.20) for being included in the multivariate model building.

In the final multivariate regression model (Table 5), work ability showed the highest odds ratio (3.369, 95% CI 2.040–5.565, *p* < 0.001) and had an explanatory value, Nagelkerke R Square, of 0.177. The Nagelkerke R Square value is a pseudo R-square value that demonstrates how well the model explains the dependent variable from 0 to 1. Aerobic capacity added 0.063 to the Nagelkerke R Square value of the total model, mental fatigue added another 0.028, residential community added 0.021 and physical fatigue added 0.007. The final model had a total Nagelkerke R Square value of 0.296 (*p* < 0.001) (*n* = 619).

## 4. Discussion

The aim of this study was to investigate perceived consequences regarding everyday life, health, physical activity and work post-COVID-19 and factors associated with low life satisfaction. We found that a majority of our sample with post-COVID-19 experienced physical fatigue, mental fatigue, dizziness, balance impairments, reduced aerobic capacity and work ability. In addition, most perceived low satisfaction with Life as a whole, and all but one of the domain-specific items of LiSat-11 showed a higher proportion with low satisfaction relative to reference values. Poor work ability was the most important factor for perceiving low overall life satisfaction. Reduced aerobic capacity, mental fatigue, living in a city and physical fatigue were factors that also increased the odds of experiencing low life satisfaction in post-COVID-19.

The results of the present study showed that post-COVID-19 may persist long after recovery from the acute COVID-19 infection, even in this sample of younger patients with milder initial infection. The most commonly reported consequence of post-COVID-19 was fatigue (both physical and mental fatigue), which is in accordance with previous studies [13,14,15,16]. In addition, dizziness was commonly experienced as well as balance impairments which also have been reported in previous studies, probably due to the involvement of vestibular and visual systems in SARS-CoV-2 infections [13,14,47].

The most strenuous activity that could be sustained for at least 30 min was walking or cycling slowly (median RPC = 5), which is considerably lower compared to levels of physical activity reported in a Swedish study among adults during the first wave of the pandemic [48]. In that study, the mean RPC was 11.5, i.e., being able to run for at least 30 min. Reduced levels of physical activity have been found in previous studies of post-COVID-19 [14,15]. It was also found that physical exertion can cause a worsening or relapse of symptoms [13,14]. In contrast, it has been suggested that physical exercise may alleviate the sequelae of COVID-19 through the release of circulating factors that mediate the anti-inflammatory response and support brain homeostasis [49]. Long-term sequelae and difficulties in regaining required levels of physical activity in this relatively young population may lead to longer-term health risks of inactivity. Therefore, more research is needed to recommend and pace rehabilitation interventions regarding physical activity for persons with post-COVID-19.

Furthermore, our results showed that many of the participants perceived reduced work ability. This is in line with a previous study where a majority of the respondents with remaining COVID-19 symptoms reported working fewer hours or were in need of a reduced work schedule. In addition, many perceived that the worsening of COVID-19 symptoms could be triggered by stress and mental exertion at work [13]. This emphasizes the importance of work capacity assessments, workplace adjustments and consideration of returning to work in relation to recovery from COVID-19.

A majority (77%) of our participants perceived low satisfaction with Life as a whole, and almost all responded that they perceived a deterioration in life satisfaction due to COVID-19. The percentage of low satisfaction in the present study was considerably higher than in the Swedish reference sample [22] and in community-dwelling persons during the first wave of the COVID-19 pandemic in Sweden [26] and also compared to people with chronic conditions such as stroke [17], traumatic brain injuries [50] and Parkinson’s disease [51]. For the domain-specific items in the current study, all but one showed a significantly higher proportion of persons perceiving low satisfaction with life compared to the Swedish reference sample [22]. Many of our participants (>70%) experienced low satisfaction with Physical health, Leisure, Intimacy, Psychological health, Contacts with friends and Vocation. For items of close relations, such as Family life and Partner relationship, half of the participants perceived low satisfaction. Our results suggest that the participants perceived more difficulties in engaging in activities and social contexts outside the family, which is reasonable to expect as many of the participants perceived fatigue and had difficulties managing their work. In addition, the pandemic restrictions might also have contributed to more difficulties in being able to participate in usual leisure activities and social events.

Economy was the only domain-specific item of LiSat-11 that did not differ significantly in the proportion of persons with low satisfaction in comparison with the reference values [22]. This might be due to the fact that most persons were working and that the social security system in Sweden provides for persons on sick leave. Thus, the participants might not have been economically affected in this stage, but in a longer perspective, the financial situation may be a more important issue.

In the multivariate regression model, poor work ability showed the highest odds of perceiving low satisfaction with Life as a whole. This result demonstrates the importance of work for well-being. In a recent cohort study, workplace modifications have been found to be the most important factor in supporting the return to work for persons with post-COVID-19 [52]. Therefore, in the future, focus should be given to how a return to work and stay at work can be supported after COVID-19.

Aerobic capacity and mental and physical fatigue were also included in our final logistic model. Those who reported lower aerobic capacity and a high level of fatigue generally had higher odds of perceiving low satisfaction with Life as a whole. However, the explanatory values of these variables were relatively low, probably due to their relation to work ability. Adapted rehabilitation interventions focusing on increasing physical activity levels and reducing fatigue may have a positive effect on life satisfaction post-COVID-19.

The logistic regression model also showed that persons living in cities compared to less densely populated areas in towns and villages had higher odds of perceiving low life satisfaction. In previous studies, similar findings have demonstrated that a higher population density can affect life satisfaction negatively [24,25]. The reason may be that larger cities are associated with anonymity and poorer neighborhood quality, as well as less support from family and contact with friends [25].

Interestingly, comorbidity was not significantly associated with low life satisfaction, despite the fact that comorbidity is a risk factor for developing post-COVID-19 and that comorbidity might increase post-COVID-19 [9]. An explanation for this may be that persons with chronic conditions are used to adapting life to fluctuations in their disease [53], while healthy persons might not expect long-term sequelae and have less experience in handling such situations. As there is little information and resources in the healthcare system to support people with post-COVID-19, there is also a risk that their symptoms have been diminished or ignored [54]. Such psychological aspects might contribute to the perception of low life satisfaction in people with post-COVID-19. More knowledge is thus needed to further understand the patient experience of post-COVID-19 in order to be able to address these issues in rehabilitation programs.

### 4.1. Strengths and Limitations

A strength of the current study was that validated questionnaires were used to assess the perceived consequences of post-COVID-19, and the large sample size allowed for the use of multivariate regression analyses. However, the study has some limitations that should be regarded when interpreting the results. By recruiting via social media, the study attracted a selected group of mostly well-educated women that are more frequent on Facebook, Instagram, etc. Nevertheless, the fact that more women participated may partly be because post-COVID-19 is twice as common in women younger than 60 [55,56]. Furthermore, there is a possibility of recall bias and subjective rating of symptoms in this type of survey. Moreover, the participants in the current study did not have to prove a test-verified COVID-19 infection. However, it has been shown that symptoms do not differ between persons who have tested positive for COVID-19 infection and those who have not been tested but show suggested symptoms [13]. Moreover, it cannot be excluded that other factors may be of importance for life satisfaction, such as cognitive and emotional functions, support from healthcare and social services, as well as socioeconomic status and cultural background.

### 4.2. Study Implications

This study has important healthcare implications. As people with post-COVID-19 report a wide range of long-term consequences, this may have a large impact on their return to normal life, including previous levels of physical activity and work. Recovery may also be negatively affected by stress and exertion that can worsen or cause a relapse of symptoms. In addition, our findings imply that post-COVID-19 has a major negative impact on general well-being. Post-COVID-19 may, therefore, increase the burden on the healthcare system and also have a wider economic impact on society. Thus, people with long-term consequences post-COVID-19 may need comprehensive assessments of their physical and cognitive function, ability to manage daily life, work and quality of life. Person-centered multidisciplinary rehabilitation ought to be provided by healthcare professionals that have a thorough understanding of the post-COVID-19 condition and knowledge of how to optimize recovery.

## 5. Conclusions

This study has shown that physical and mental fatigue, dizziness, balance impairments, reduced aerobic capacity, poor work ability and low life satisfaction are commonly perceived consequences of post-COVID-19. Work ability, aerobic capacity and fatigue are factors associated with low life satisfaction. The results of this study can serve as guidance for healthcare authorities regarding important long-term consequences that should be considered in rehabilitation programs directed toward post-COVID-19. Future studies should focus on how post-COVID-19 consequences change over time and evaluate the efficacy of rehabilitation protocols for persons with post-COVID-19.

## Figures and Tables

**Table 1:** Characteristics of the study sample (*n* = 766).

Variable	Values
Age (*n* = 766)	
Mean (SD; range)	48 (10; 18–80)
Age groups (*n* = 766)	
<30, % (*n*)	4 (33)
30–45, % (*n*)	38 (287)
46–60, % (*n*)	48 (364)
60+, % (*n*)	11 (82)
Sex (*n* = 757)	
Men, % (*n*)	11 (83)
Women, % (*n*)	89 (672)
Family situation (*n* = 758)	
Single, % (*n*)	21 (160)
Married/cohabiting, % (*n*)	74 (567)
Partner, not cohabiting, % (*n*)	5 (39)
Residential community (*n* = 763)	
City, % (*n*)	32 (242)
Town, % (*n*)	30 (228)
Village, % (*n*)	38 (293)
Educational level (*n* = 764)	
Primary (8–9 years), % (*n*)	1 (9)
Secondary (10–12 years), % (*n*)	27 (203)
Higher education (college/university), % (*n*)	72 (552)
Provision (*n* = 765)	
Work, % (*n*)	69 (526)
Student grants, % (*n*)	3 (23)
Sickness benefit, % (*n*)	17 (132)
Unemployment benefit, % (*n*)	1.5 (12)
Social security benefit, % (*n*)	0.5 (3)
Other sources of income, % (*n*)	9 (69)
Comorbidities (*n* = 764)	
Yes, % (*n*)	39 (301)
Duration of post-COVID-19 (*n* = 766)	
Mean months (SD; range)	13 (SD 5; 2–25)

**Table 2:** Perceived consequences of post-COVID-19.

Variable	Values
Physical fatigue (FFS) (*n* = 732)	
Total score (0–7), median (IQR)	6.0 (4.9–6.7)
Score ≥ 4, % (*n*)	85 (624)
Mental Fatigue (MFS) (*n* = 699)	
Total score (0–42), median (IQR)	18 (12–22.5)
Score ≥ 10.5, % (*n*)	84 (586)
Aerobic capacity (RPC) (*n* = 676)	
Score (1–20), median (IQR)	5 (3–7)
Dizziness (*n* = 691)	
Yes, % (*n*)	84 (578)
Balance impairment (*n* = 699)	
Yes, % (*n*)	56 (391)
Daily activities (ADL Staircase) (*n* = 665)	
P-ADL score (0–15), median (IQR)	0 (0–0)
I-ADL score (0–15), median (IQR)	1 (0–5)
Total score (0–30), median (IQR)	2 (0–5)
Work ability (WAS) (*n* = 625)	
Score (0–10), median (IQR)	5 (2–7)
Poor (0–5 points), % (*n*)	52 (324)
Moderate (6–7 points), % (*n*)	26 (164)
Good (8–9 points), % (*n*)	18 (114)
Excellent (10 points), % (*n*)	4 (23)

**Table 3:** Life satisfaction and LiSat-11 scores in persons with post-COVID-19.

Items of LiSat-11	Median (IQR)	Low Satisfaction, % (*n*)	Reference Value (%)	*p*-Value *
1. Life as a whole (*n* = 650)	4 (3–4)	77 (502)	30	<0.001
2. Vocation (*n* = 643)	4 (2–5)	72 (463)	46	<0.001
3. Economy (*n* = 647)	4 (3–5)	58 (378)	61	0.096
4. Leisure (*n* = 645)	3 (2–4)	89 (534)	43	<0.001
5. Contacts with friends (*n* = 646)	4 (2–5)	75 (481)	35	<0.001
6. Intimacy (*n* = 628)	3 (1–4)	79 (495)	44	<0.001
7. ADL (*n* = 643)	5 (4–6)	33 (214)	5	<0.001
8. Family life (*n* = 628)	4 (4–5)	51 (318)	19	<0.001
9. Partnership (*n* = 556)	5 (4–5)	48 (265)	18	<0.001
10. Physical health (*n* = 647)	3 (2–4)	91 (587)	28	<0.001
11. Psychological health (*n* = 648)	4 (3–4)	75 (489)	19	<0.001
Perceived change in Life as a whole (*n* = 646)				
Improved, % (*n*)		1 (6)		
Unchanged, % (*n*)		12 (78)		
Deteriorated, % (*n*)		87 (562)		
Perceived deterioration in Life as a whole related to COVID-19 (*n* = 562)				
Yes, % (*n*)		82 (462)		
Partly, % (*n*)		16 (89)		

IQR: Inter Quartile Range. Reference value: proportion of persons with low satisfaction with Life as a whole in the Swedish reference sample (based on ratings of 2533 individuals aged 18 to 65 years) according to Fugl-Meyer et al. [22]. * Comparison to reference sample by One Sample Proportion Test.

**Table 4:** Univariate logistic regression analyses of factors associated with low satisfaction with Life as a whole in persons with post-COVID-19.

Variables	Odds Ratio (95% CI)	Nagelkerke R Square	*p*-Value
Sociodemographic factors			
Age	0.990 (0.973–1.008)	0.003	0.280
Sex (men vs. ref women)	1.182 (0.649–2.152)	0.001	0.585
Family situation (single vs. ref married/partner)	2.294 (1.345–3.911)	0.025	0.002
Educational level (lower vs. ref higher education)	1.290 (0.842–1.974)	0.003	0.242
Provision (not working vs. ref working)	2.396 (1.528–3.757)	0.037	<0.001
Residential community (city vs. ref town/village)	2.329 (1.484–3.655)	0.035	<0.001
Comorbidities (ref no)	1.207 (0.825–1.766)	0.002	0.333
Perceived consequences of COVID-19			
Physical fatigue, FSS (fatigue vs. ref no fatigue)	6.608 (4.103–10.641)	0.135	<0.001
Mental fatigue, MFS (fatigue vs. ref no fatigue)	5.791 (3.717–9.921)	0.134	<0.001
Dizziness (dizziness vs. ref no dizziness)	2.063 (1.322–3.220)	0.022	<0.001
Balance impairment (impairment vs. ref no impairment)	1.949 (1.450–2.825)	0.029	<0.001
Daily activity, ADL staircase score	1.223 (1.140–1.312)	0.003	<0.001
Aerobic capacity, RPC score	0.769 (0.719–0.823)	0.153	<0.001
Work ability, WAS (poor vs. ref moderate–excellent ability)	6.255 (3.978–9.837)	0.177	<0.001

Life as a whole obtained by item 1 in LiSat-11. CI: confidence interval. Nagelkerke R Square: pseudo R-square value that demonstrates how well the model explains the dependent variable (from 0 to 1). Ref: reference in the logistic regression analysis for nominal variables.

**Table 5:** Multivariate logistic regression analyses of factors associated with low satisfaction with Life as a whole in persons with post-COVID-19 (*n* = 619).

Variables	Odds Ratio (95% CI)	*p*-Value
Work ability, WAS (poor vs. ref moderate–excellent ability)	3.369 (2.040–5.565)	<0.001
Aerobic capacity, RPC score	0.860 (0.796–0.929)	<0.001
Mental fatigue, MFS (fatigue vs. ref no fatigue)	2.049 (1.148–3.657)	0.015
Residential community (city vs. ref town/village)	2.208 (1.334–3.657)	0.002
Physical fatigue, FSS (fatigue vs. ref no fatigue)	1.844 (0.982–3.461)	0.057
Total model: Nagelkerke R Square 0.296 (*p*-value < 0.001)		

## Data Availability

The data used in this study contain sensitive information about the study participants, and they did not provide consent for public data sharing. The current approval by the Swedish Ethical Review Authority (Dnr 2020-02776) does not include data sharing. A minimal data set could be shared by request from a qualified academic investigator for the sole purpose of replicating the present study, provided the data transfer is in agreement with EU legislation on the general data protection regulation and approval by the Swedish Ethical Review Authority.
